# Supplementary material for: Machine Learning Accelerates Crystallization for Structure Determination
Source: Angew Chem Int Ed Engl. 2026 May 4;65(25):e1218503. doi: 10.1002/anie.1218503 (PMC13266958; doi:10.1002/anie.1218503)

checkCIF/PLATON report

Structure factors have been supplied for datablock(s) lcz083

THIS REPORT IS FOR GUIDANCE ONLY. IF USED AS PART OF A REVIEW PROCEDURE FOR PUBLICATION, IT SHOULD NOT REPLACE THE EXPERTISE OF AN EXPERIENCED CRYSTALLOGRAPHIC REFEREE.

No syntax errors found. CIF dictionary Interpreting this report

Datablock: lcz083

```
Bond precision:   C-C = 0.0123 A          Wavelength=1.54184

Cell:            a=16.3878(15)    b=13.2075(13)      c=16.0469(17)
                 alpha=90         beta=91.059(9)     gamma=90
Temperature:     100 K
```

	Calculated	Reported
Volume	3472.6(6)	3472.6(6)
Space group	I 2/a	I 1 2/a 1
Hall group	-I 2ya	-I 2ya
Moiety formula	C29 H13 Ag3 F18 N8 O2	C15 H3 Ag3 F18 N6, C14 H10 N2 O2
Sum formula	C29 H13 Ag3 F18 N8 O2	C29 H13 Ag3 F18 N8 O2
Mr	1171.08	1171.08
Dx,g cm-3	2.240	2.240
Z	4	4
Mu (mm-1)	14.764	14.764
F000	2248.0	2248.0
F000’	2258.68	
h,k,lmax	20,16,19	20,16,19
Nref	3511	3324
Tmin,Tmax	0.086,0.094	0.510,1.000
Tmin’	0.009	

```
Correction method= # Reported T Limits: Tmin=0.510 Tmax=1.000
AbsCorr = MULTI-SCAN
```

Data completeness= 0.947   Theta(max)= 73.434

R(reflections)= 0.0533(2742)   wR2(reflections)= 0.1403(3324)

S = 1.069   Npar= 329

The following ALERTS were generated. Each ALERT has the format
test-name_ALERT_alert-type_alert-level.
Click on the hyperlinks for more details of the test.

Alert level C

```
PLAT042_ALERT_1_C Calc. and Reported MoietyFormula Strings  Differ     Please Check
              Calc: C29 H13 Ag3 F18 N8 O2
              Rep.: C15 H3 Ag3 F18 N6, C14 H10 N2 O2
PLAT213_ALERT_2_C Atom F1               has ADP max/min Ratio .....        3.6 prolat
PLAT213_ALERT_2_C Atom F00D             has ADP max/min Ratio .....        3.6 prolat
PLAT215_ALERT_3_C Disordered O18        has ADP max/min Ratio .....        3.2 Note
PLAT342_ALERT_3_C Low Bond Precision on  C-C Bonds ...............    0.01229 Ang.
PLAT767_ALERT_4_C INS Embedded LIST 6 Instruction Should be LIST 4     Please Check
PLAT906_ALERT_3_C Large K Value in the Analysis of Variance ......      3.182 Check
PLAT911_ALERT_3_C Missing FCF Refl Between Thmin & STh/L=    0.600         46 Report
              -16  9  1,  10  9  1,  15 10  1, -16  8  4, -18  5  5, -15  9  6,
               18  0  6, -15  8  7,  18  1  7, -18  0  8,  16  0  8,  17  1  8,
               16  1  9,  17  2  9,  13  1 10,  14  0 10,  15  1 10,  16  0 10,
               16  2 10,   0 13 11,  14  1 11,  15  2 11,  15  4 11,  -4 12 12,
               12  0 12,  13  1 12,  14  0 12,  14  2 12,  14  4 12,  15  1 12,
                   (   16 More Missing: see the .ckf listing file)
PLAT972_ALERT_2_C Check Calcd Resid. Dens.  1.00Ang From Ag01          -1.59 eA-3
PLAT972_ALERT_2_C Check Calcd Resid. Dens.  0.87Ang From Ag02          -1.57 eA-3
PLAT972_ALERT_2_C Check Calcd Resid. Dens.  0.87Ang From Ag01          -1.51 eA-3
```

Alert level G

```
PLAT002_ALERT_2_G Number of Distance or Angle Restraints on AtSite          8 Note
PLAT003_ALERT_2_G Number of Uiso or U(i,j)  Restrained non-H-Atoms         18 Report
PLAT007_ALERT_5_G Number of Unrefined Donor-H Atoms .............           4 Report
              H9A   H9B   H10A  H10B
PLAT083_ALERT_2_G SHELXL Second Parameter in WGHT  Unusually Large      54.65 Why ?
PLAT172_ALERT_4_G The CIF-Embedded .res File Contains DFIX Records          2 Report
PLAT178_ALERT_4_G The CIF-Embedded .res File Contains SIMU Records          1 Report
PLAT188_ALERT_3_G A Non-default SIMU Restraint Value has been used     0.0100 Report
PLAT242_ALERT_2_G Low    'MainMol' Ueq as Compared to Neighbors of       C00L Check
PLAT242_ALERT_2_G Low    'MainMol' Ueq as Compared to Neighbors of       C00M Check
PLAT242_ALERT_2_G Low    'MainMol' Ueq as Compared to Neighbors of       C00Q Check
PLAT299_ALERT_4_G Atom Site Occupancy Constrained at ............         0.5 Check
              O1      O18     N9      N10     C2      C3      C4      C5
              C6      C7      C8      C11     C12     C13     C14     C15
              C16     C17     H5      H7      H9A     H9B     H10A    H10B
              H11     H12     H13     H14
PLAT301_ALERT_3_G Main Residue  Disorder ..............(Resd    1)        30% Note
PLAT434_ALERT_2_G Short Inter HL..HL Contact F1        ..F00D     .      2.82 Ang.
                                                   x,-1+y,z  =      1_545 Check
PLAT720_ALERT_4_G Number of Unusual/Non-Standard Labels .........          21 Note
```

```
              Ag01    Ag02    F003    F004    F006    F007    N008    F009
              N00A    F00B    F00D    C00E    H00E    C00G    C00H    C00I
              C00K    H00K    C00L    C00M    C00Q
PLAT789_ALERT_4_G Atoms with Negative _atom_site_disorder_group  #         28 Check
PLAT811_ALERT_5_G No ADDSYM Analysis: Too Many Excluded Atoms ....          ! Info
PLAT822_ALERT_4_G CIF-embedded .res Contains Negative PART Numbers          3 Check
PLAT860_ALERT_3_G Number of Least-Squares Restraints ............         126 Note
PLAT912_ALERT_4_G Missing # of FCF Reflections Above STh/L=  0.600        141 Note
PLAT933_ALERT_2_G Number of HKL-OMIT Records in Embedded .res File          3 Note
               12  4 14,  12  6 14,  11  4 15,
PLAT941_ALERT_3_G Average HKL Measurement Multiplicity ..........         3.7 Low
PLAT969_ALERT_5_G The 'Henn et al.' R-Factor-gap value ..........       3.667 Note
              Predicted wR2: Based on SigI**2  3.83 or SHELX Weight 13.13
PLAT978_ALERT_2_G Number C-C Bonds with Positive Residual Density.          0 Info
```

```
   0 ALERT level A = Most likely a serious problem - resolve or explain
   0 ALERT level B = A potentially serious problem, consider carefully
  11 ALERT level C = Check. Ensure it is not caused by an omission or oversight
  23 ALERT level G = General information/check it is not something unexpected

   1 ALERT type 1 CIF construction/syntax error, inconsistent or missing data
  14 ALERT type 2 Indicator that the structure model may be wrong or deficient
   8 ALERT type 3 Indicator that the structure quality may be low
   8 ALERT type 4 Improvement, methodology, query or suggestion
   3 ALERT type 5 Informative message, check
```

Validation response form

Please find below a validation response form (VRF) that can be filled in and pasted into your CIF.

```
# start Validation Reply Form
_vrf_PLAT042_lcz083
;
PROBLEM: Calc. and Reported MoietyFormula Strings  Differ     Please Check
RESPONSE: ...
;
_vrf_PLAT213_lcz083
;
PROBLEM: Atom F1              has ADP max/min Ratio .....        3.6 prolat
RESPONSE: ...
;
_vrf_PLAT215_lcz083
;
PROBLEM: Disordered O18       has ADP max/min Ratio .....        3.2 Note
RESPONSE: ...
;
_vrf_PLAT342_lcz083
;
PROBLEM: Low Bond Precision on  C-C Bonds ..............    0.01229 Ang.
RESPONSE: ...
;
_vrf_PLAT767_lcz083
;
PROBLEM: INS Embedded LIST 6 Instruction Should be LIST 4     Please Check
RESPONSE: ...
```

```
;
_vrf_PLAT906_lcz083
;
PROBLEM: Large K Value in the Analysis of Variance ......      3.182 Check
RESPONSE: ...
;
_vrf_PLAT911_lcz083
;
PROBLEM: Missing FCF Refl Between Thmin & STh/L=    0.600         46 Report
RESPONSE: ...
;
_vrf_PLAT972_lcz083
;
PROBLEM: Check Calcd Resid. Dens.  1.00Ang From Ag01           -1.59 eA-3
RESPONSE: ...
;
# end Validation Reply Form
```

It is advisable to attempt to resolve as many as possible of the alerts in all categories. Often the minor alerts point to easily fixed oversights, errors and omissions in your CIF or refinement strategy, so attention to these fine details can be worthwhile. It is up to the individual to critically assess their own results and, if necessary, seek expert advice.

PLATON version of 17/09/2025; check.def file version of 17/09/2025

---

duplicate check

No duplication found

Datablock lcz083 - ellipsoid plot

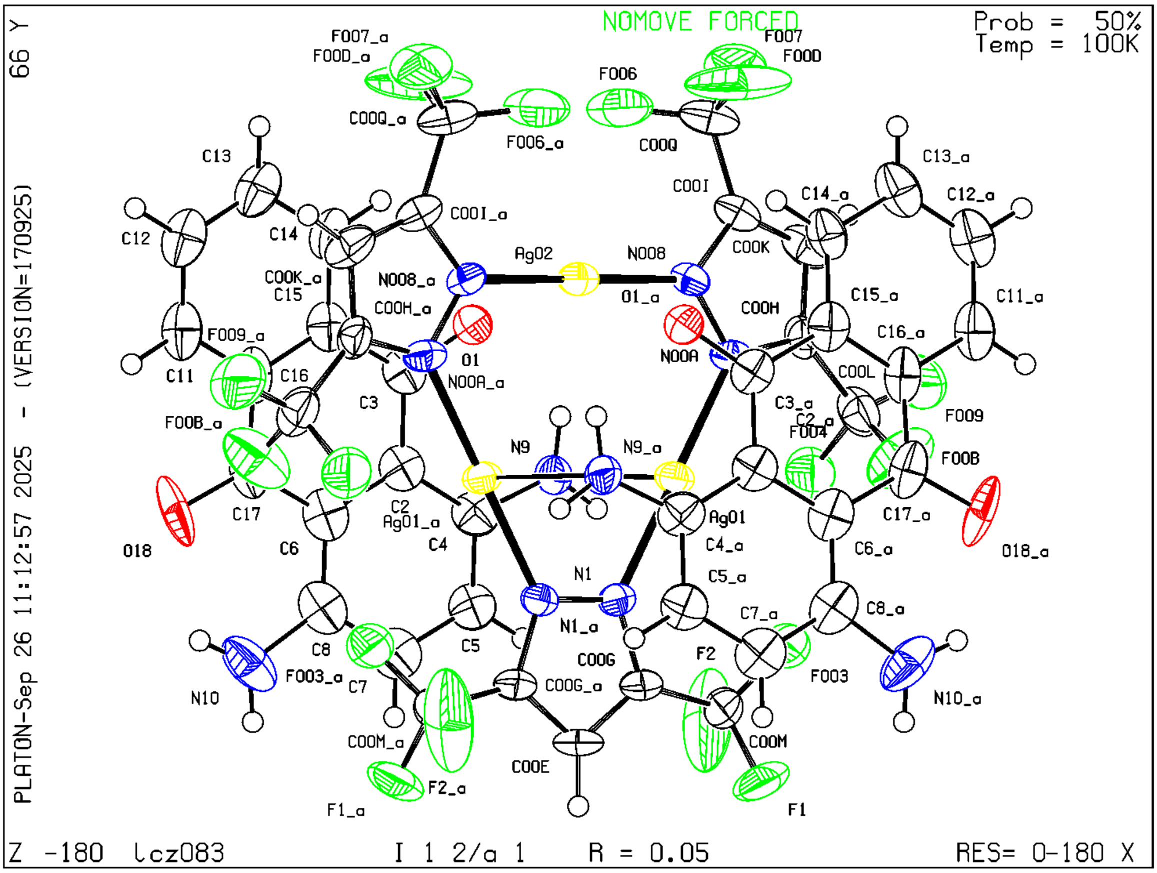

Supplement: Supplementary file 2 — Supporting File 2: anie72462‐sup‐0002‐DataFile.zip. [file ANIE-65-e1218503-s002.zip › supplementary_materials_crystallographic_data submit.zip!/cif_and_checkcif(1-114)/Ag3Pz3_83/Ag3Pz3_83_checkcif.pdf]
